# Effects of probiotic supplementation on the anthropometric nutritional status of patients with type 2 *Diabetes mellitus*: A systematic review and meta-analysis protocol

**DOI:** 10.1371/journal.pone.0314971

**Published:** 2024-12-06

**Authors:** Dálete Assíria de Souza Ribeiro, Anny Cristine de Araújo, Priscila Kelly da Silva Bezerra do Nascimento, Ilanna Marques Gomes da Rocha, Adriana Augusto de Rezende

**Affiliations:** 1 Nutrition Postgraduate Program, Center for Health Sciences, Federal University of Rio Grande Do Norte, Natal, Brazil; 2 Health Sciences Postgraduate Program, Center for Health Sciences, Federal University of Rio Grande do Norte, Natal, Brazil; 3 Department of Gastroenterology, Faculty of Medicine, Hospital das Clinicas HCFMUSP, University of São Paulo, São Paulo, Brazil; 4 Department of Clinical and Toxicological Analyses, Federal University of Rio Grande do Norte, Natal, Brazil; Università degli Studi di Milano, ITALY

## Abstract

**Background:**

*Diabetes mellitus* (DM) is characterized by hyperglycemia due to insufficient insulin production or utilization. Previous studies have shown a relationship between the gut microbiota and DM, driving interest in probiotic supplementation to modulate the microbiota and glucose metabolism in patients with DM, although the exact mechanisms remain unclear. Probiotics can influence metabolic factors and improve the composition of the microbiota, possibly helping to reduce weight in patients with DM.

**Objective:**

The objective of this review is to compile and analyze the most relevant evidence on the effects of probiotic supplementation on the nutritional anthropometric status of patients with type 2 Diabetes mellitus (T2DM).

**Methods:**

Methodological guidelines will be followed in accordance with the Preferred Reporting Items for Systematic Reviews and Meta-Analyses statement and the study has been registered in the International Prospective Register of Systematic Reviews under reference number CRD42023480243. Studies will be selected through an active search of the PubMed, Science Direct, and SCOPUS databases using the following search descriptors: gut microbiota, body weight, and metabolic diseases, according to medical subject headings. The assessment of the methodological quality of the studies will be carried out using the Cochrane Collaboration instrument. The risk of bias will be analyzed using the Revised Cochrane tool for risk of bias in randomized controlled trials (RoB 2). A meta-analysis will be performed if heterogeneity is acceptable and justifiable; otherwise, the results will be presented in a qualitative narrative synthesis.

**Expected results:**

The results of probiotic supplementation are expected to demonstrate improvements in anthropometric parameters such as body weight, BMI and abdominal and waist circumference in patients with T2DM, thus providing valuable evidence for clinical application.

## Introduction

*Diabetes mellitus* (DM) is a chronic disease with a significant impact on the global health system. Estimates from the International Diabetes Federation indicate that globally, 537 million adults between 20 and 79 years of age have DM, and this is projected to reach 643 million by 2030 and 783 million by 2045 [1].

Type 2 *Diabetes mellitus* (T2DM) is the most prevalent type of diabetes, accounting for more than 90% of all DM cases globally [1]. The disease is multifactorial, with strong genetic and environmental influences. Obesity and an unhealthy lifestyle are the main risk factors for its high incidence [2]. Weight control is a recommended therapeutic strategy for preserving glucose and insulin function and mitigating the progression of chronic diseases in patients with T2DM [3, 4].

Difficulty in controlling weight contributes to the development of overweight, a condition widely associated with elevated levels of inflammation, which exacerbates metabolic disorders in T2DM [5] and promotes the onset of intestinal dysbiosis. Dysbiosis, characterized by alterations in the composition of the gut microbiota, has been correlated with dysfunctions in energy homeostasis regulation [6].

In this context, probiotics have emerged as a promising therapeutic strategy to restore gut microbiota balance. Moreover, these microorganisms exhibit versatile anti-obesity effects, largely attributed to their production of short-chain fatty acids (SCFAs), which modulate hormones involved in appetite regulation, improve insulin sensitivity, and increase energy expenditure [7, 8]. Particularly noteworthy is the synthesis of conjugated linoleic acid, which plays a role in mechanisms conducive to weight reduction. These mechanisms include lipid oxidation, adipocyte apoptosis, and the suppression of lipogenesis and inflammation [9].

Probiotics can alter the composition of gut bacteria, leading to increased production of SCFA that play a critical role in energy and appetite regulation. For instance, a recent meta-analysis found that probiotics could significantly reduce body weight and BMI, especially in overweight and obese populations, suggesting potential therapeutic benefits for managing weight in metabolic disorders like T2DM [10]. Another systematic review demonstrated that specific strains, such as *Lactobacillus* and *Bifidobacterium*, contribute to reduced fat accumulation and improved lipid metabolism, which are essential factors in controlling body weight [11].

Although probiotic supplementation is suggested to help reduce weight in individuals with T2DM and recommended as a complementary therapeutic intervention [12], the specific effects of probiotics in preventing obesity in this population are not yet fully understood.

Therefore, the objective of this proposed study is to establish an accurate methodology to evaluate, through a systematic review, the effects of probiotic supplementation on the complete nutritional status of individuals with T2DM. This assessment will include not only weight or BMI, but also other measurements, such as abdominal and waist circumference, aiming for a more comprehensive analysis of nutritional status based on anthropometric parameters. By investigating the potential effects of probiotics in this population, we can obtain valuable knowledge about the way that probiotics may contribute to the development of novel complementary therapeutic approaches.

## Methods

The review protocol was developed following the guidelines of the Preferred Reporting Items for Systematic Reviews and Meta-Analyses Protocol (PRISMA-P) [13] (S1 File). It is reported in the International Prospective Register of Systematic Reviews (PROSPERO) under reference number CRD42023480243. The guidelines of the PRISMA statement [14] were used to prepare this systematic review protocol.

### Ethics

Ethical approval is not required as this systematic review will be based on previously published data. The obtained results will be submitted for publication in a peer-reviewed scientific journal and updated if new evidence emerges that can modify the conclusions.

### PICOS strategy

The PICOS strategy used in the review question is shown in Table 1 [15].

**Table 1:** Definition of the research question structured in the PICOS format.

Acronym	Definition	Description
P	Population	Patients with T2DM;
I	Intervention	Supplementation of probiotics at any dose (CFU/g), form of administration and lasting more than one week;
C	Control	Participant with T2DM who did not receive supplementation or received placebo;
O	Outcome	Anthropometric measurements: body weight (kg), BMI (kg/m^2^), waist perimeter (cm) and abdominal perimeter (cm).
S	Study design	Randomized clinical trial

To formulate the structured question, we used the PICOS strategy (P: population, I: intervention, C: comparison, O: outcome, S: study design), which is commonly used in clinical trials [15]. The diagnosis of T2DM was made based on several parameters related to blood glucose levels: fasting blood glucose (FBG) ≥ 126 mg/dL; blood glucose concentration 2 hours after ingesting 75 g of glucose (oral glucose tolerance test) ≥ 200 mg/dL; glycated hemoglobin (HbA1c) ≥ 6.5%; and random glycemia > 200 mg/dL [16].

To diagnose overweight and obesity, authors should adhere to the guidelines outlined by the World Health Organization [17]. According to these guidelines, individuals with a BMI ranging from 25 kg/m ^2^ to 30 kg/m^2^ are classified as overweight, whereas those with a BMI exceeding 30 kg/m^2^ are categorized as obese.

The following anthropometric measurements will be analyzed as the primary outcomes in this systematic review: BMI (kg/m^2^), body weight (kg), waist perimeter (cm), and abdominal perimeter (cm). These measures are widely used because of their simplicity, low cost, immediate availability, and patient understanding. Administration of probiotics may play a beneficial role in reducing body weight and anthropometric measurements in individuals with obesity and diabetes, thus constituting a potentially effective complementary therapeutic strategy for this patient population.

#### Inclusion criteria

Randomized clinical trials carried out in patients with T2DM alone that investigate the effects of probiotic supplementation on nutritional status will be considered eligible, regardless of the strains and dosages used. Eligible studies will be required to report at least one of the following anthropometric measurements: body weight, BMI, waist perimeter and abdominal perimeter. Participants aged 18–75 years will be included in the study. Only articles in English will be included and there will be no restrictions on the publication period of the studies. These criteria follow the PICOS structure presented in Table 1.

#### Exclusion criteria

Studies will be excluded if patients have other comorbidities associated with DM, if they have less than 1 week of follow-up duration; do not have a placebo or control group; are carried out on pregnant or lactating women; do not provide sufficient information on the previously mentioned outcome indicators; are summaries, case reports, study protocols, review articles, or letters, or do the full text is not available; or are cross-sectional, cohort, or case-control observational studies. Duplicate data reported in multiple studies will also be excluded. Furthermore, studies investigating the combined effects of probiotics with other vitamins and/or nutritional supplements or the simultaneous use of prebiotics and probiotics will be excluded from consideration.

#### Search strategy

The terms analyzed will include intestinal microbiota, anthropometric measurements, and T2DM. The search algorithm will use terms registered on the Medical Subject Headings (MeSH) platform, which is widely used in scientific articles, using Boolean operators. Table 2 presents an example search strategy applied to the PubMed database. The full search strategy for MEDLINE is provided in S2 File.

**Table 2:** Search strategy used for PubMed.

Search terms	
1	*Diabetes mellitus*, Type II
2	*Diabetes mellitus* adult onset
3	*Diabetes mellitus*, Noninsulin-Dependent
4	Type 2 Diabetes
5	*Diabetes mellitus* type II
6	Hyperglycemia
7	OR / 1–7
8	Dietary supplements
9	Food Supplements
10	Probiotics
11	Gastrointestinal microbiome
12	Gut microbiome
13	Microflora gut
14	Intestinal microbiota
15	Gastrointestinal microbiomes
16	Enteric bactéria
17	Human microbiome
18	Microbial communities
19	Community composition microbial
20	Cultured milk products
21	Fermented Dairy Products
22	Fermented Milk Products
23	Yogurt
24	Kefir
25	Kefir Grains
26	OR / 8–26
27	Body mass index
28	Index body mass
29	Quetelet s index
30	Anthropometry
31	Waist circumference
32	Body size
33	Body weight
34	Glycemic control
35	Blood glucose control
36	Signs and symptoms, digestive
37	Cholesterol
38	Triglycerides
39	Triglyceride
40	Triacylglycerols
41	Interleukin 6
42	IL-6
43	Tumor necrosis factor alpha
44	TNF-alpha
45	C reactive protein
46	hs-CRP
47	High sensitivity c reactive protein
48	OR/ 27–48
49	Clinical trial
50	Clinical study
51	7 AND 26 AND 48

The search will be conducted using the following electronic bibliographic databases: ScienceDirect, Scopus, and PubMed. The search strategy will include terms related to PICOS. A search of the “gray literature” will be conducted using Google Scholar [18].

There will be no restrictions on the publication date, however articles only in English published until September 2024 will be included. The filter will be applied to identify only those studies conducted in humans. All results will be organized using Rayyan QCRI software [19].

A manual search of the records will be conducted to identify relevant references in the articles included in the review.

### Data selection and analysis

**Fig 1:**
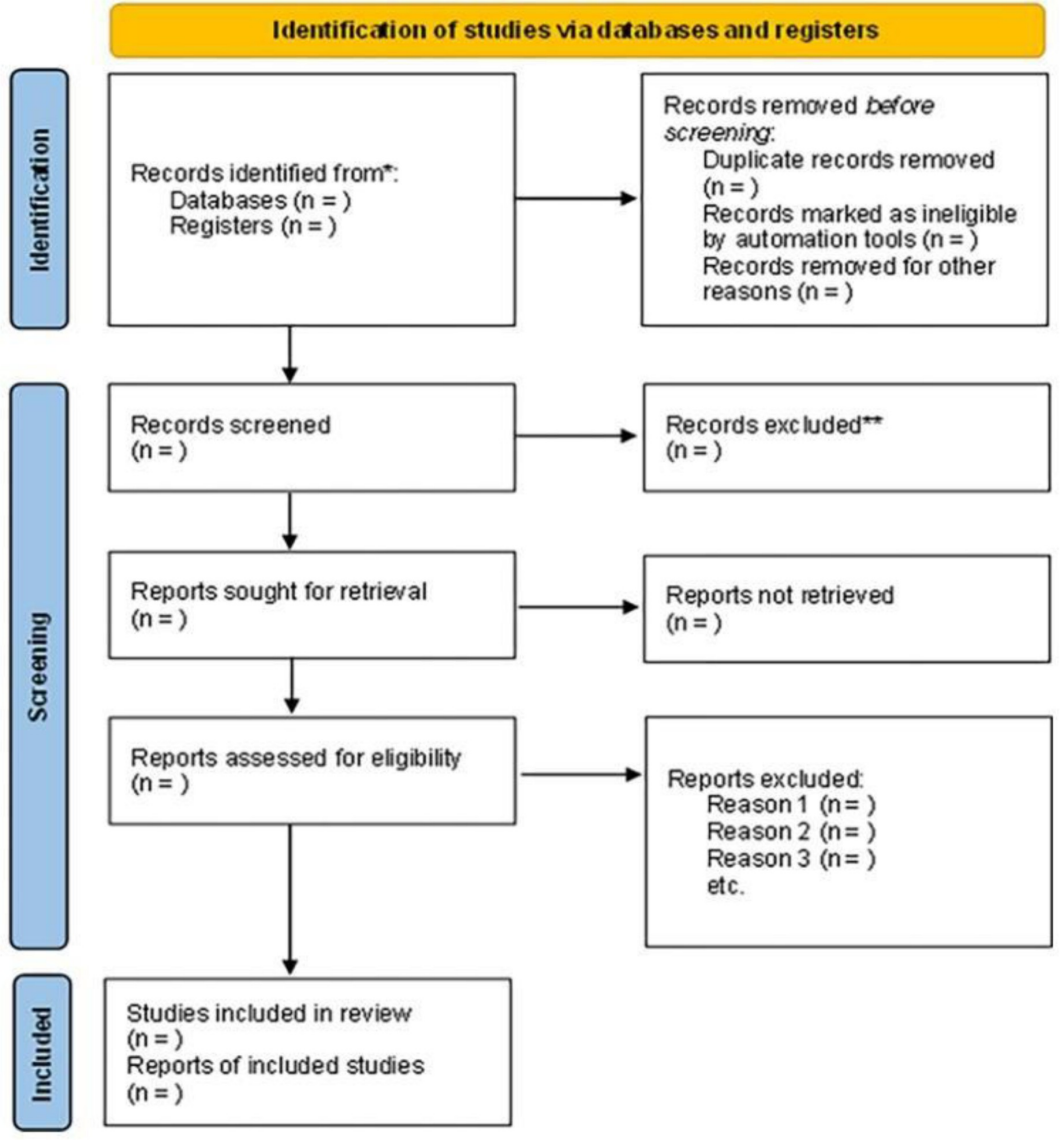
Flow diagram for systematic review and meta-analysis. PRISMA 2020.

The selection and screening of studies will be carried out independently by three reviewers (DASR, ACA, and PKSBM), with titles and abstracts analyzed against the eligibility criteria. This step will be carried out using Rayyan QCRI software [19], which is used in systematic reviews to read titles and abstracts and remove duplicate articles. The same reviewers will independently access the full texts of the selected studies. Only the studies that meet the eligibility criteria will be included in this review, after the complete text is read. During the review stages, the reasons for excluding studies will be recorded, and a fourth reviewer (AAR) will be consulted to resolve any differences in the evaluations. During the review phase, the reasons for excluding studies will be noted. Fig 1 illustrates the study selection process.

The expected start date for the selection of articles is December 2024, and the expected completion date is January 2025.

### Data extraction

Three reviewers (DASR, ACA, and PKSBN) will independently extract and cross-check data from all eligible studies. In case of divergent opinions, a fourth reviewer (AAR) will be consulted for resolution. The extracted data will cover the study characteristics (including first author, year of publication, location, recruitment period); participant characteristics (such as average age, sex, number of participants, medication); details about probiotic supplementation (type, dose, duration, route of administration); study quality risk assessment; glycemic control: fasting glycemia, HbA1c levels (glycated hemoglobin), postprandial glycemia, HOMA-IR (Assessment of the homeostasis model of insulin resistance), lipid profile: total cholesterol, low-density lipoprotein-C, high-density lipoprotein-C, triglyceride levels, and side effects: nausea, vomiting, abdominal pain, intestinal constipation, diarrhea. Additionally, improvements in gastrointestinal symptoms; the levels of inflammatory markers, such as C-reactive protein, tumor necrosis factor alpha, and interleukin-6; and alterations in the gut microbiota composition will be evaluated. These changes may encompass changes in the diversity and abundance of specific bacterial species.

Data from each included study will be extracted and organized in a table (Microsoft Excel) (S3 File).

### Missing data

In dealing with missing data, we will make efforts to recover any missing data by contacting the lead or corresponding author, as well as the co-authors of the article, via telephone, email, or postal mail. If the necessary information is not obtained, the data will be excluded from our analysis and discussed in the appropriate section.

### Assessment of methodological quality

The risk of bias will be assessed independently by three reviewers, using the Revised Cochrane tool for risk of bias in randomized controlled trials (RoB 2) [20]. Any discrepancies will be resolved through discussion with a fourth reviewer.

RoB 2 is structured into five domains: (1) Randomization process; (1.1) Timing of identification or recruitment of participants in cluster randomized trials (applicable only to this type of trial); (2) Deviations from planned interventions; (3) Missing outcome data; (4) Assessment of outcomes; and (5) Selection of reported results. For each domain, signaling questions are categorized as "yes", "probably yes", "probably no", "no", or "no information". An algorithm is then used to determine the risk of bias for each domain, classified as "low risk", "some concerns" or "high risk of bias". After this step, the study’s overall risk of bias is determined based on the following criteria: low risk (in all domains); some concerns (in at least one domain, without high risk in any); or high risk of bias (in at least one domain or multiple concerns across multiple domains) [20].

Three reviewers (DASR, ACA, and PKSBM) will resolve disagreements by consensus; however, if necessary, a fourth reviewer (AAR) will participated in the decision.

### Risk of bias

Publication bias will be evaluated if more than 10 studies have been included with a funnel plot. To assess the asymmetry of the funnel plots, Egger’s test will be performed when more than 10 studies are included. In addition, Egger’s weighted correlation and Begg’s regression intercept at a 5% significance level will be conducted [21]. To identify and correct for funnel plot asymmetry due to publication bias, the trim and fill technique will be performed [22].

### Data synthesis

For the meta-analysis, quantitative synthesis will be performed using STATA software (STATA Corp, College Station, TX, USA). The inverse variance method, applying the random-effects model, will be used if the heterogeneity between the studies exceeds 50%.

For dichotomous variables, the odds ratios (ORs) and 95% confidence intervals (CIs) (fixed-effects model) will be extracted or calculated. For continuous variables, the difference in weighted means will be calculated (random effects model) with a corresponding 95% CI.

Heterogeneity among trial results will be evaluated using the standard I^2^ statistic, which quantifies the percentage of variation in results across studies. An I^2^ value near 0% indicates no observed heterogeneity, while values approaching 25%, 50%, and 75% correspond to low, moderate, and high heterogeneity, respectively. In cases where heterogeneity is identified (I^2^ > 50%), a random-effects model will be employed to aggregate studies and calculate the odds ratio (OR) with a 95% CI. Conversely, if heterogeneity is low (I^2^ < 50%), a fixed-effects model will be applied. Forest plots will be generated to illustrate study-specific and pooled relative risk/OR estimates [23].

Meta-analysis will not be conducted under circumstances of extreme and unexplained heterogeneity (I^2^ >75% without sufficient justification), irreconcilable methodological differences, incompatible outcomes among studies, an inadequate number of studies, poor methodological quality, significant publication bias, or insufficient or inconsistent data. Therefore, a narrative synthesis will be undertaken to describe the individual characteristics of each study while emphasizing qualitative similarities, differences, and implications.

### Subgroup and sensitivity analyses

Sensitivity analysis will be performed to identify significant changes in the estimated prevalence and to evaluate the 95% CIs. This study will aim to identify potential sources of heterogeneity by excluding low-quality studies.

If relevant, analyses will be carried out considering the following subgroups: age; sex; BMI; number of probiotic strains; and the presence of confounding factors, such as environmental factors, smoking status, and alcohol consumption. Significantly different results between subgroups (*p* < 0.05) will be reported individually, and a test will be employed to evaluate these interactions using STATA software.

### Assessment of the certainty of evidence

To assess the certainty of the evidence, the Grading of Recommendations Assessment, Development and Evaluation (GRADE) [24] approach will be used to assess the strength of the evidence of the results of the systematic review. The GRADE tool classifies studies as low, moderate, or high quality. Three reviewers (DASR, ACA, and PKSBM) will carry out this assessment regardless of any disagreements, and the decision will be made through discussion with the fourth reviewer (AAR).

The factors responsible for the reduction in the level of evidence will be classified as methodological limitations (risk of bias), inconsistency, indirect evidence, imprecision, and publication bias.

A summary of the assessment will be incorporated into a table with the result of the judgment of each factor that alters the certainty of the evidence and the result of the quality assessment of that outcome.

## Discussion

Here, we provide a clear and transparent methodology for conducting a systematic review and meta-analysis to investigate the effects of probiotic supplementation on anthropometric variables and the nutritional status of patients with T2DM.

Previous research has highlighted the intricate relationship between T2DM and the gut microbiota. Typically, individuals with T2DM exhibit a diminished presence of SCFA-producing bacteria, which are known to regulate appetite and energy intake by stimulating satiety hormones. These bacteria include *Eubacterium rectale*, *Faecalibacterium prausnitzii*, *Roseburia intestinalis*, *Akkermansia muciniphila*, and *Bifidobacterium* spp. [25–27]. Conversely, obese individuals often demonstrate altered gut microbiota composition, including a reduction in bacterial richness and diversity, along with an increase in the number of microorganisms with pro-inflammatory characteristics. Thus, probiotic supplementation is posited as a means to modulate the microbiota and potentially enhance weight loss outcomes [6].

Although a 5% reduction in body weight is accepted as significant weight loss, there is some evidence to suggest that a smaller percentage of weight loss may be beneficial. The exclusive use of probiotics may not be clinically effective for weight loss; however, probiotic supplementation can be introduced as a safe complementary approach to common weight loss strategies, such as calorie restriction and increased physical activity [28].

A randomized, double-blind, placebo-controlled study carried out in patients with T2DM for six months observed a significant reduction in the waist-to-hip ratio in the group that received synbiotic supplementation after 12 weeks of intervention [29]. Furthermore, another study was carried out with 43 participants also with T2DM for 8 weeks to investigate the effects of a synbiotic on body weight, BMI and blood glucose. The results demonstrated a significant reduction in weight and BMI in the group that received the supplementation, compared to the placebo group [30]. However, it is important to highlight that these studies focused on symbiotic supplementation and the last study did not include other anthropometric variables, such as abdominal and waist circumference.

A systematic review with meta-analyses was conducted with the aim of investigating the impact of probiotic supplementation on body weight in patients with T2DM. The pooled data indicated that there were no significant differences between the probiotic group and the placebo group [31]. However, BMI was the only anthropometric measurement considered. Furthermore, the study had significant methodological limitations, including an insufficient search strategy and the inclusion of studies that used probiotics in combination with other interventions in the final analyses.

Despite the controversy surrounding the effects of probiotics on the nutritional status of patients with T2DM, this systematic review aims to address this gap by offering objective evidence for the impact of probiotic supplementation on the anthropometric characteristics of patients with T2DM. Therefore, the possible regulation of the intestinal microbiota aims to prevent colonization by pathogenic bacteria, remodel the composition of the commensal microbiota, reduce the production of pro-inflammatory cytokines, and strengthen the intestinal barrier. These effects can positively benefit the anthropometric characteristics of these patients. This evidence has the potential to guide doctors and nutritionists in choosing more effective treatments, mitigating excessive weight gain, and improving the clinical results and quality of life of affected individuals.

The potential limitations of this study include study characteristics, such as sample size, duration, efficacy of different probiotic strains, inappropriate statistical analysis, and inadequate reporting of primary study methods and results. Regarding BMI, the anthropometric measurements used do not reflect the distribution of body fat. To mitigate these limitations, the team responsible for the review is committed to using rigorous methodology to systematically identify and thoroughly analyze studies that are aligned with the objectives of the review.

In this context, this proposed review will provide evidence and insights relevant to everyday clinical practice, with the aim of improving the quality and effectiveness of interventions. These probiotic supplements, which are characterized by favorable safety profiles, have a positive effect on the metabolic functions of patients with T2DM. In this way, the results of the study will improve nutritional assistance for patients diagnosed with T2DM, through the development of new strategies that promote the reversal of obesogenic processes, in addition to enabling the identification of innovative treatments.

## Supporting information

S1 File PRISMA-P 2015 checklist. (DOCX)

S2 File Search strategy in different databases. (DOCX)

S3 File Standard form for data extraction. (XLSX)
