# Supplementary material for: Effects of probiotic supplementation on the anthropometric nutritional status of patients with type 2 Diabetes mellitus: A systematic review and meta-analysis protocol
Source: PLoS One. 2024 Dec 6;19(12):e0314971. doi: 10.1371/journal.pone.0314971 (PMC11623458; doi:10.1371/journal.pone.0314971)
Supplement: S2 File — (DOCX) [file pone.0314971.s002.docx]

**S2 File. Search strategy in different databases.**

| **Base de dados** | **Estratégia** | **Resultados** |
| --- | --- | --- |
| *Pubmed* | "diabetes mellitus, type 2"[MeSH Terms] OR "diabetes mellitus adult onset"[Title/Abstract] OR "diabetes mellitus noninsulin dependent"[Title/Abstract] OR "type 2 diabetes"[Title/Abstract] OR "diabetes mellitus type ii"[Title/Abstract] OR "hyperglycemia"[MeSH Terms] AND "dietary supplements"[MeSH Terms] OR "Food Supplements"[Title/Abstract] OR "probiotics"[MeSH Terms] OR "gastrointestinal microbiome"[MeSH Terms] OR "gut microbiome"[Title/Abstract] OR "microflora gut"[Title/Abstract] OR "intestinal microbiota"[Title/Abstract] OR "gastrointestinal microbiomes"[Title/Abstract] OR "enteric bacteria"[Title/Abstract] OR "Microbiota"[MeSH Terms] OR "human microbiome"[Title/Abstract] OR "microbial communities"[Title/Abstract] OR "community composition microbial"[Title/Abstract] OR "cultured milk products"[MeSH Terms] OR "Fermented Dairy Products"[Title/Abstract] OR "Fermented Milk Products"[Title/Abstract] OR "yogurt"[MeSH Terms] OR "Kefir"[MeSH Terms] OR "Kefir Grains"[Title/Abstract] AND "body mass index"[MeSH Terms] OR "index body mass"[Title/Abstract] OR "quetelet s index"[Title/Abstract] OR "anthropometry"[MeSH Terms] OR "waist circumference"[MeSH Terms] OR "body size"[MeSH Terms] OR "body weight"[MeSH Terms] OR "glycemic control"[MeSH Terms] OR "blood glucose control"[Title/Abstract] OR "signs and symptoms, digestive"[MeSH Terms] OR "cholesterol"[MeSH Terms] OR "triglycerides"[MeSH Terms] OR "Triglyceride"[Title/Abstract] OR "Triacylglycerols"[Title/Abstract] OR "interleukin 6"[MeSH Terms] OR "IL-6"[Title/Abstract] OR "tumor necrosis factor alpha"[MeSH Terms] OR "TNF-alpha"[Title/Abstract] OR "TNFalpha"[Title/Abstract] OR "c reactive protein"[MeSH Terms] OR "c reactive protein"[Title/Abstract] OR "hs-CRP"[Title/Abstract] OR "high sensitivity c reactive protein"[Title/Abstract] AND "clinical trial"[Publication Type] OR "clinical study"[Publication Type] | 411 |
| *Science direct* | ("Diabetes Mellitus, Type 2'') AND (Probiotics OR "dietary supplements" OR "Fermented Dairy Products") AND (anthropometry OR "body mass index" OR "Glycemic Control") AND ("Clinical Trial" OR "clinical study") | 162 |
| *Scopus* | ((TITLE-ABS-KEY ( diabetes AND mellitus, AND type 2 ) AND TITLE-ABS-KEY ( probiotics ) OR TITLE-ABS-KEY ( dietary AND supplements ) OR TITLE-ABS-KEY ( gastrointestinal AND microbiome ) OR TITLE-ABS-KEY ( cultured AND milk AND products ) OR TITLE-ABS-KEY ( fermented AND dairy AND products ) OR TITLE-ABS-KEY ( fermented AND milk AND products ) OR TITLE-ABS-KEY ( yogurt ) OR TITLE-ABS-KEY ( kefir ) AND TITLE-ABS-KEY ( body AND mass AND index ) OR TITLE-ABS-KEY ( anthropometry ) OR TITLE-ABS-KEY ( waist AND circumference ) OR TITLE-ABS-KEY ( body AND weight ) OR TITLE-ABS-KEY ( glycemic AND control ) OR TITLE-ABS-KEY ( cholesterol ) OR TITLE-ABS-KEY ( triglycerides ) OR TITLE-ABS-KEY ( interleukin-6 ) OR TITLE-ABS-KEY ( tumor AND necrosis AND factor-alpha ) OR TITLE-ABS-KEY ( c-reactive AND protein ) AND TITLE-ABS-KEY ( clinical AND trial )) | 885    5680 |
|  | **LITERATURA CINZENTA** |  |
| *Google Acadêmico* | (("type 2 diabetes" OR hyperglycemia) AND (probiotic OR "Gut Microbiome" OR "microflora gut") AND (anthropometry OR "Abdominal Circumference") AND (Cholesterol OR triglycerides OR "Glycemic Control" OR "tumor necrosis factor alpha") AND ("Clinical Trial" OR "clinical study")) |  |
